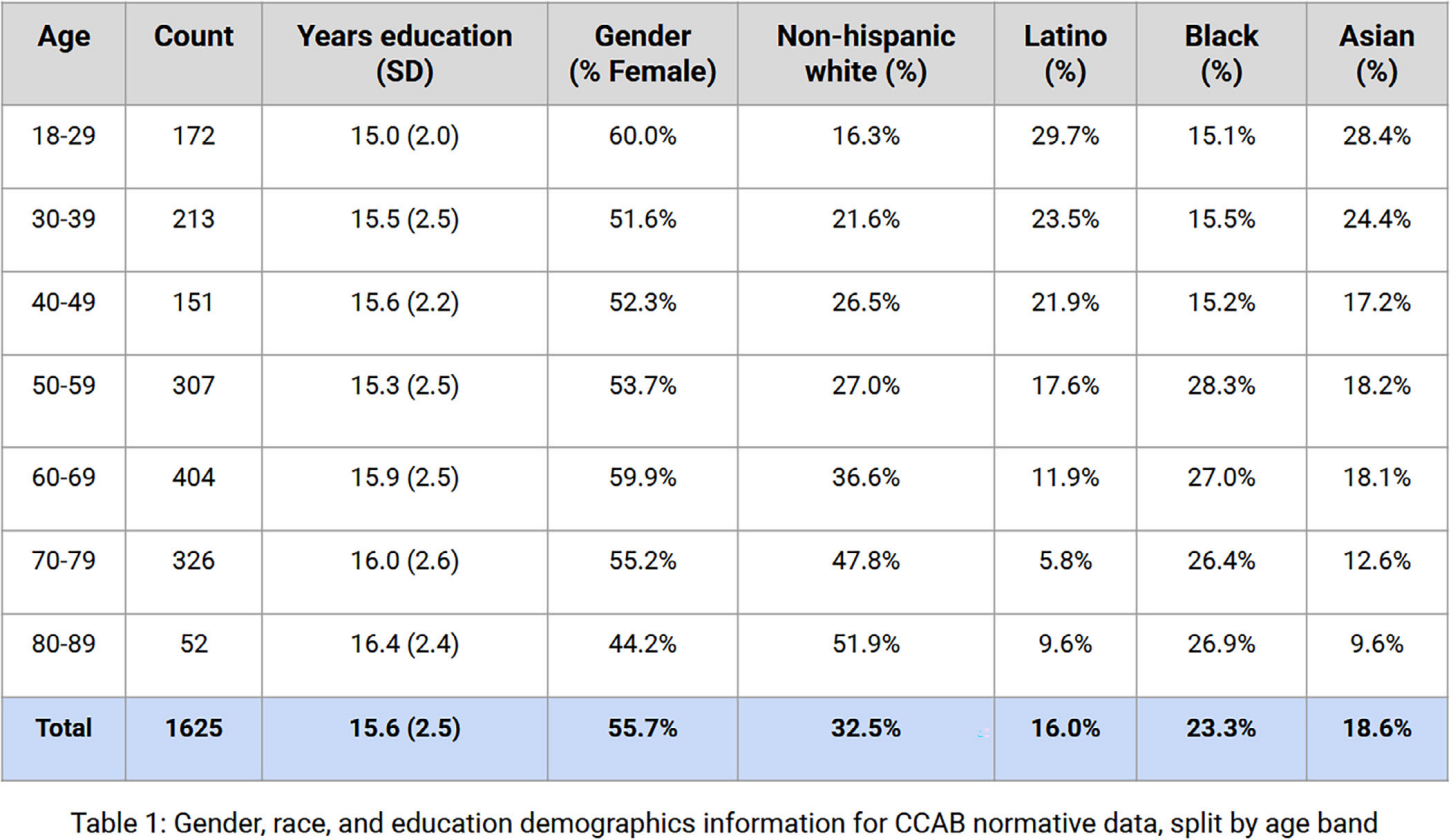# A large public dataset of computerized cognitive test results with high‐resolution speech quantification

**DOI:** 10.1002/alz70861_108426

**Published:** 2025-12-23

**Authors:** Michael Blank, Kathleen Hall, Kristin Geraci, David K Johnson, Timothy J Herron, Peter Pebler, Omar Kahly, David L. Woods

**Affiliations:** ^1^ Neurobehavioral Systems, Inc, Berkeley, CA USA; ^2^ UC Davis Alzheimer's Disease Center, Walnut Creek, CA USA; ^3^ Veterans Affairs Northern California Health Care System, Martinez, CA USA

## Abstract

**Background:**

We present a publicly available normative dataset with results from the California Cognitive Assessment Battery (CCAB), to support research into cognition, aging, and speech and language biomarkers. This dataset is hosted on Open Science Framework (OSF) to facilitate collaborative and/or independent secondary analyses by other researchers.

**Method:**

1,625 participants (55.7% female, 37% white; mean age 55.2 ± 17.0 years; see Table 1) completed the CCAB normative protocol. Most completed ∼6 total hours of supervised testing across three consecutive days, with identical tests administered on days 2 and 3 to measure retest learning effects; others completed a single two‐hour test session. Some participants also completed follow‐up assessments at one (*N* =480), two (*N* =244), and three (*N* =174) years post‐enrollment. More than 95% of participants were tested in their homes using telemedical proctoring.

CCAB is an automated, computerized test battery with 22 verbal and 18 non‐verbal tasks, administered on calibrated hardware with Presentation® software for sub‐millisecond temporal precision. CCAB tasks span multiple cognitive domains and response modalities, including touch (e.g., trail making), mouse (e.g., choice reaction time), and speech (e.g., picture description). Extended demographic and health data were also collected, including psychological scales (e.g., GDS, CFQ, GAD‐7), a vocabulary‐based estimate of premorbid intelligence, and lifestyle and medical history.

For speech tasks, audio recordings were transcribed using consensus automatic speech recognition (CASR), which combines multiple ASR engines to achieve >98% word‐level transcription accuracy. High‐quality recordings and high‐accuracy automated transcriptions enable fine‐grained quantification of speech timing, fluency, syntax, and phonetics.

**Result:**

Each individual test run (N > 100,000) was analyzed to produce summary performance metrics such as category‐wise response counts, speech complexity measures, response timing, and speech acoustics. Results were collated by task, deidentified, and paired with metadata and data dictionaries following OSF best practices, and are available on OSF.

**Conclusion:**

We invite researchers to explore and further analyze this large, high‐resolution dataset for studies in neuropsychology, speech‐language processing, longitudinal cognition, or machine learning. We will continue to update the OSF data repository (https://osf.io/x8u5z/) with new and more granular data.